# A survey of RNA editing at single-cell resolution links interneurons to schizophrenia and autism

**DOI:** 10.1261/rna.078804.121

**Published:** 2021-12

**Authors:** Brendan Robert E. Ansell, Simon N. Thomas, Roberto Bonelli, Jacob E. Munro, Saskia Freytag, Melanie Bahlo

**Affiliations:** 1 Population Health and Immunity Division, Walter and Eliza Hall Institute of Medical Research, Parkville 3052, Victoria, Australia; 2 Department of Medical Biology, University of Melbourne, Parkville 3052, Victoria, Australia; 3 Molecular Medicine Division, Harry Perkins Institute of Medical Research, Nedlands 6009, Western Australia, Australia

**Keywords:** RNA editing, RNA recoding, cerebral cortex, neuron, SMART-seq, single-nucleus sNuc-seq, nucleolus, Prader–Willi locus, small nucleolar RNA, Alu repeat, differential editing, epilepsy, autism spectrum disorder, schizophrenia

## Abstract

Conversion of adenosine to inosine in RNA by ADAR enzymes, termed “RNA editing,” is essential for healthy brain development. Editing is dysregulated in neuropsychiatric diseases, but has not yet been investigated at scale at the level of individual neurons. We quantified RNA editing sites in nuclear transcriptomes of 3055 neurons from six cortical regions of a neurotypical female donor, and found 41,930 sites present in at least ten nuclei. Most sites were located within Alu repeats in introns or 3′ UTRs, and approximately 80% were cataloged in public RNA editing databases. We identified 9285 putative novel editing sites, 29% of which were also detectable in unrelated donors. Intersection with results from bulk RNA-seq studies provided cell-type and spatial context for 1730 sites that are differentially edited in schizophrenic brain donors, and 910 such sites in autistic donors. Autism-related genes were also enriched with editing sites predicted to modify RNA structure. Inhibitory neurons showed higher overall transcriptome editing than excitatory neurons, and the highest editing rates were observed in the frontal cortex. We used generalized linear models to identify differentially edited sites and genes between cell types. Twenty nine genes were preferentially edited in excitatory neurons, and 43 genes were edited more heavily in inhibitory neurons, including *RBFOX1*, its target genes, and genes in the autism-associated Prader–Willi locus (15q11). The abundance of SNORD115/116 genes from locus 15q11 was positively associated with editing activity across the transcriptome. We contend that insufficient editing of autism-related genes in inhibitory neurons may contribute to the specific perturbation of those cells in autism.

## INTRODUCTION

The extraordinary structural and functional complexity of the human brain arises via multiple mechanisms of genetic regulation. The conversion of adenosine to inosine (A > I) in nascent RNA transcripts in the nucleolus by ADAR1 and ADAR2 enzymes, known as “RNA editing,” is the most abundant RNA modification in the primate central nervous system, and confers transcriptomic diversity beyond that encoded in the genome (Slotkin and Nishikura 2013). RNA editing is essential for healthy brain development and increases with age (Hwang et al. 2016).

Dysregulated editing is implicated in epilepsy (Srivastava et al. 2017), glioblastoma (Silvestris et al. 2019), major depression (Lyddon et al. 2013), autism spectrum disorder (Tran et al. 2019), and schizophrenia (Breen et al. 2019). ADAR1 primarily edits adenosine within repetitive sequence regions; ADAR2 primarily edits nonrepetitive sequences in brain tissue, and ADAR3 is a catalytically inactive inhibitor of editing (Li et al. 2009b). ADAR2-null mice die in utero, and partial knockout animals succumb to severe seizures soon after birth (Higuchi et al. 2000). In humans, mutations in ADAR1 cause skin dyschromatosis (MIM 127400), and Aicardi-Goutieres encephalopathy (MIM 615010) with clinical subtypes including striatal and motor neurodegeneration (Livingston et al. 2014; Gallo et al. 2017). Mislocalization of ADAR2 was recently reported in human and mouse models of C9orf72-mediated amyotrophic lateral sclerosis (ALS) (Moore et al. 2019). Common SNPs in the ADAR gene family have also been implicated in numerous traits and diseases including hippocampal volume, intellectual disability, microcephaly, epilepsy (Tan et al. 2020), type II diabetes, and aspects of Alzheimer's disease and lung cancer (Buniello et al. 2019).

Recently thousands of edited sites were identified in bulk RNA sequencing of human tissues including several brain regions (Tan et al. 2017) as part of the GTEx consortium projects (GTEx Consortium 2020). The most well-characterized edited site is an A > I conversion at exonic nucleotide 2135 of the glutamate receptor subunit transcript *GLUR2*, which produces a Q > R amino acid substitution. This site is edited in nearly 100% of human *GLUR2* transcripts, and limits the calcium permeability of the resulting ion channel, which is thought to dampen neuronal excitability (Nishikura 2006). Unlike this “gold standard” *GLUR2* site, most editing sites are located in noncoding regions of the transcriptome, particularly within Alu repeats that form double-stranded RNA on which ADAR enzymes act. Editing of noncoding regions can nevertheless affect protein expression via intron retention, splice site variation and altered translation efficiency (Zhou et al. 2013).

The RNA editing landscape has been described both in the neurotypical brain and in brains from subjects with neurological and neuropsychiatric illnesses. However, results to date derive from bulk RNA sequencing data, which while informative, leaves much to be discovered about the regional and cellular specificity of this process in single cells. Unlike gene expression analysis, RNA editing analysis requires greater sequencing depth and transcript coverage, which is more expensive to generate than widely used short 3′-targeted single-cell sequencing protocols. Picardi and colleagues (2017b) previously profiled editing rates at protein recoding sites in 268 brain cell nuclei from seven adult and three foetal brains, and found a markedly bimodal distribution of edited allele frequency in contrast to the continuous distribution reported in bulk tissue. This suggests that bulk RNAseq-based editing measurements belie highly penetrant editing events restricted to certain cell-types or tissue regions, and are thus the average of binary signals which may depend on cell type origin. To better understand RNA editing dynamics in single brain cells, and to detect novel sites that may be subsumed in bulk RNA sequencing, we compared RNA editing in more than 3000 single neurons from six cortical regions of the left hemisphere from an individual donor, previously reported by Lake et al. (2016). This data was generated using the SMART-seq full-length transcript capture platform and is enriched for nuclear RNA, thus allowing deep insight into editing of noncoding and pre-mRNAs in cortical neurons of the healthy adult brain. We applied rigorous statistical methods to identify clinical and transcriptomic correlates of RNA editing in this data set, and report differential site- and gene editing across neuronal subtypes and cortical regions. This work represents the largest and most comprehensive analysis of RNA editing in single cells of any biological system to date.

## RESULTS

In the first part of the results we describe the characteristics of edited sites across the entire population of neuronal nuclei. We then report differential editing (dEd) analyses between cortical regions, cell types, and genes therein. The final section reports correlation analyses between gene abundance and editing rates that identify potential modulators of neuronal RNA editing.

### Thousands of novel putative RNA editing sites revealed at single-cell resolution

Our independent read processing and unsupervised clustering of 3127 neuronal nuclei from a single donor, originally assayed by Lake et al. (2016), separated nuclei according to the neuronal subtypes determined by the study authors (Supplemental Fig. S1). We discarded 72 nuclei due to either high mitochondrial reads (*n* = 42), or low library complexity (*n* = 30) (Supplemental Fig. S2). We therefore quantified RNA editing signals in 3055 high-quality neuronal nuclear transcriptomes, supported by 9.17bn uniquely mapped reads. Excitatory neurons from the superior temporal cortex (Brodmann area 41) were the most abundantly represented cell type in this data set (606 cells; 29.7% of total). The mean ratio of inhibitory to excitatory neurons across each cortical region was 0.285, or approximately 1 inhibitory to every 3 excitatory neurons.

**FIGURE 1.**
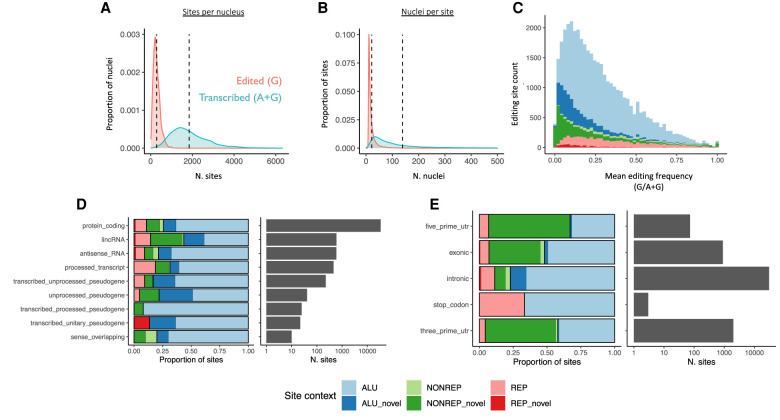
Distribution of 41,930 RNA editing sites across neuronal nuclei, transcriptional frequency, gene biotypes, and features. Density of edited and transcribed (including unedited) sites detected (*A*) per nucleus (*x*-axis), and (*B*) across neuronal nuclei (“nuclei per site” on *x*-axis). Dashed lines indicate the series mean. (*C*) Distribution of mean minor (edited) allele frequencies for cataloged (light hues) and novel sites (dark hues) located within Alu repeats (blues); non-Alu repeats (reds) and nonrepetitive sequence (greens). Individual density plots are provided in Supplemental Figure S4b. (*D*) Distribution of sites colored by site context, across RNA biotypes (*y*-axis). Number of sites is indicated by bars on *right* panel, on a log scale. (*E*) Distribution of protein-coding sites across gene features (*y*-axis), with number of sites indicated by bars in *right* panel.

Adenosine editing produces an inosine base, which is represented as guanosine in RNA sequencing data. Adenosine-to-guanosine substitution was by far the most common variant detected in nuclear transcriptomes, consistent with a strong ADAR-dependent RNA editing signal relative to variants originating from common genomic SNPs (Supplemental Fig. S3). By filtering on-site coverage, prevalence, genomic context, and previous evidence, we detected 41,930 editing sites. An average of 1850 candidate editing sites were transcribed per cell (4.4% of total), of which 290 (0.7%) were edited (Fig. 1A). When site prevalence was considered, on average each site was transcribed in 138 cells (4.5% of all cells) and edited in 22 (0.7%) (Fig. 1B). The distribution of minor (G) allele frequencies within nuclei was bimodal as reported previously for a smaller set of cortical neurons (Picardi et al. 2017b). This observation was largely independent of the total read depth at edited sites (Supplemental Fig. S4a,c). When averaged across cells, the minor (i.e., edited) allele frequency distribution was positively skewed, and previously uncataloged “novel” sites showed lower overall editing frequencies (Fig. 1C; Supplemental Fig. S4b). Approximately 80% of editing sites were located within nonoverlapping regions of protein-coding genes. The majority of these (20,052; 59%) were located in intronic Alu repeats documented in the REDIportal database of human RNA editing sites (Fig. 1D; Picardi et al. 2017a). A further 4245 sites were exonic, including 3′ untranslated regions (2958 sites), 5′ UTRs (122) and stop codons (six), in broad agreement with previous genome-wide characterization of RNA editing (Fig. 1E; Li et al. 2009b).

**FIGURE 2.**
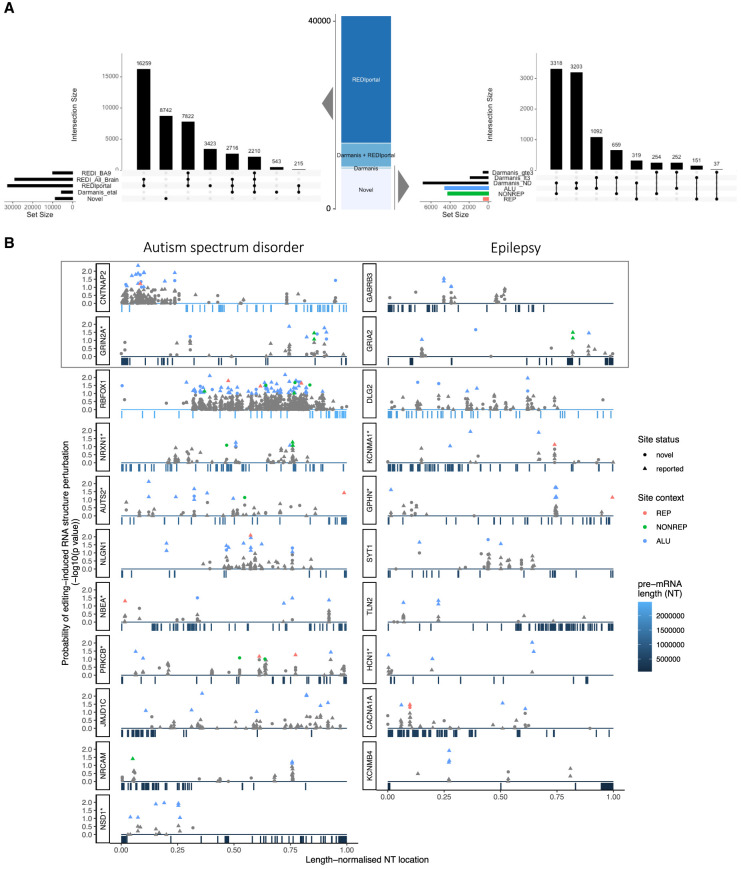
Intersection of novel and previously reported RNA editing sites from related data sets, and predicted structural perturbations in edited disease-related transcripts. (*A*) Intersection of RNA editing sites with sites detectable in other single neuron and bulk tissue data sets. (*Left*) Upset plot quantifying editing sites previously documented in the frontal cortex (BA9), whole brain, or non-CNS tissues in the REDIportal database. Sites detected in at least three of 116 neurons assayed from unrelated individuals (Darmanis et al.) are also displayed. (*Right*) Intersection of 9285 previously uncataloged, novel sites (subset of *left* panel), with sites detectable in at least three (gte3) or at least one (lt3) neurons assayed by Darmanis et al.; and their editing contexts (Alu, nonrepetitive or repetitive). *Horizontal* bars indicate set size, and *vertical* bars indicate the size of intersects displayed with linked black points in the matrix. (*B*) Location and probability of editing-induced RNA structure perturbation. Predicted effects on local RNA structure (*y*-axis) for editing sites (points) are displayed across the scaled genomic RNA coordinates (*x*-axis). The genes displayed contain at least three editing sites predicted to perturb local RNA structure, and are enriched for associations with autism spectrum disorder (*left* series) and epilepsy (*right* series). Only the editing sites with significant predicted structural effects are shown in color. Four genes implicated in both disorders are outlined in gray. *Vertical* bars *below* the *x*-axis indicate approximate exon locations, colored by relative gene length. (*) Genes with extremely low mutation tolerance.

We intersected this data with editing sites documented in 304 bulk RNA sequencing libraries from 13 brain regions (36 healthy donors) (Supplemental Table S3; Picardi et al. 2017a; Tan et al. 2017; Lo Giudice et al. 2020). Some 69.1% of all sites (*n* = 29,007) were detected in at least one bulk brain sample, of which 30.8% (10,032) were detected in the frontal cortex. A further 3638 sites were cataloged in other human tissues (Fig. 2A; Picardi et al. 2017a). Of 9285 newly detected editing sites, 4547 and 507 sites overlapped Alu repeats and non-Alu repeats respectively (Fig. 2A). The remaining 4231 novel sites were located in nonrepetitive sequence (Figs. 1D, 2A). The majority of novel sites (85%) were detected in genes containing documented RNA editing sites. Across each gene, the strongest predictor of the number of novel nonrepetitive sites was the number of novel Alu sites, which in turn was most strongly correlated with the number of documented Alu sites (Supplemental Fig. S5a). As expected, the distance between neighboring nonrepetitive sites was greater (median neighbor distance = 30 nt) than for Alu and non-Alu repetitive sites, which mostly occurred in clusters (median neighbor distance = 12 nt; Supplemental Fig. S5b). We next intersected our data with dEd sites reported from analyses of bulk brain RNA sequencing of neuropsychiatric patient cohorts, and could provide single-cell level context for 1730 of 18,071 sites edited in the frontal cortex of schizophrenic (SCZ) patients (9.5% of all SCZ-related sites) (Breen et al. 2019); and 910 of 9680 sites differentially edited in autism spectrum disorder patients (9.4% of ASD-related sites) (Tran et al. 2019). Taken together, nearly 80% of sites detected in single neuronal nuclei were previously reported in bulk transcriptome sequencing from human tissues, predominantly brain samples from both healthy and disease-affected individuals (Fig. 2A).

Given the greater sensitivity, and cell-type specificity of SMART single-nucleus RNA-seq (sNuc-seq), we sought to validate these findings in the only other publicly available sNuc-seq data from healthy human brain, containing 116 neuronal nuclei from six unrelated donors, published by Darmanis et al. (2015). Despite representing only 5% of the number of nuclei assayed by Lake and colleagues, we detected 14% of sites (5684) from the larger data set in at least three nuclei, and a further 30.5% of sites (12,781) in at least one nuclear transcriptome (Supplemental Table S3). Importantly, 26% of newly detected sites in the Lake et al. data, were also detectable in this independent sNuc-seq data set (Fig. 2A). This indicates that RNA editing in single cells is biologically conserved and can be reproducibly detected across unrelated individuals. Further, as much as 20% of editing signals detectable in single-nucleus data may derive from low-penetrance or cell type-restricted modifications that are subsumed by bulk RNA sequencing. All editing sites and associated information for the sNuc-seq data reported here are available in Supplemental Tables, and in an interactive web viewer: shiny.wehi.edu.au/ansell.b/sc_brain_browser.

### Predicted structural and functional effects of edited sites in single neurons

To investigate the relationship between RNA editing, RNA structure and genetic sequence conservation, we first investigated the predicted effect of editing sites on local RNA secondary structure. Approximately 7% of edited sites were predicted to perturb RNA structure (Supplemental Table S3). Genes containing at least three such sites were enriched for associations with autism spectrum disorder (*q* < 6 × 10^−3^), and epilepsy syndrome (*q* = 0.026), via DOSE disease enrichment analysis (Yu et al. 2015). These disease-related gene sets were in turn enriched among the 1003 human genes with the lowest tolerance for missense coding mutations (Fig. 2B; Samocha et al. 2014). We investigated the potential for missense variation conferred by RNA editing across the entire data set using the Variant Effect Predictor tool, which revealed putative amino acid substitutions in 316 cases. Missense sites were detected in seven well-established targets of ADAR enzymes that produce modified peptides upon editing, of which four (in *NEIL1*, *GRIK2*, *CYFIP2*, and the “gold standard” site in *GLUR2*) showed identical missense mutations to those previously reported (Nishikura 2016) (Supplemental Table S4). All but 12 of the 316 detected missense sites were novel, in nonrepetitive sequence regions of 223 genes that are not currently established as ADAR recoding targets. Glutamate- and aspartate-to-glycine substitutions were the most common predicted protein recoding consequences of editing at these sites (Supplemental Fig. S5c). We searched for enriched gene ontology molecular function terms among the 232 putative target genes and found both “RNA binding,” and “adenyl ribonucleotide binding” among the top 20 most enriched terms—the latter result deriving from missense sites in DEAD box helicases, HSP90 alpha AA1 and AB1, and several calcium transporting ATPases (Supplemental Fig. S5d). Together these results indicate that RNA editing preferentially affects the RNA structure of ASD and epilepsy-related genes, which as a group are less tolerant of missense genetic mutations. In contrast, editing introduces low-prevalence coding variation in genes involved in RNA processing.

### Global editing rates are higher in inhibitory neurons across cortical regions

**FIGURE 3.**
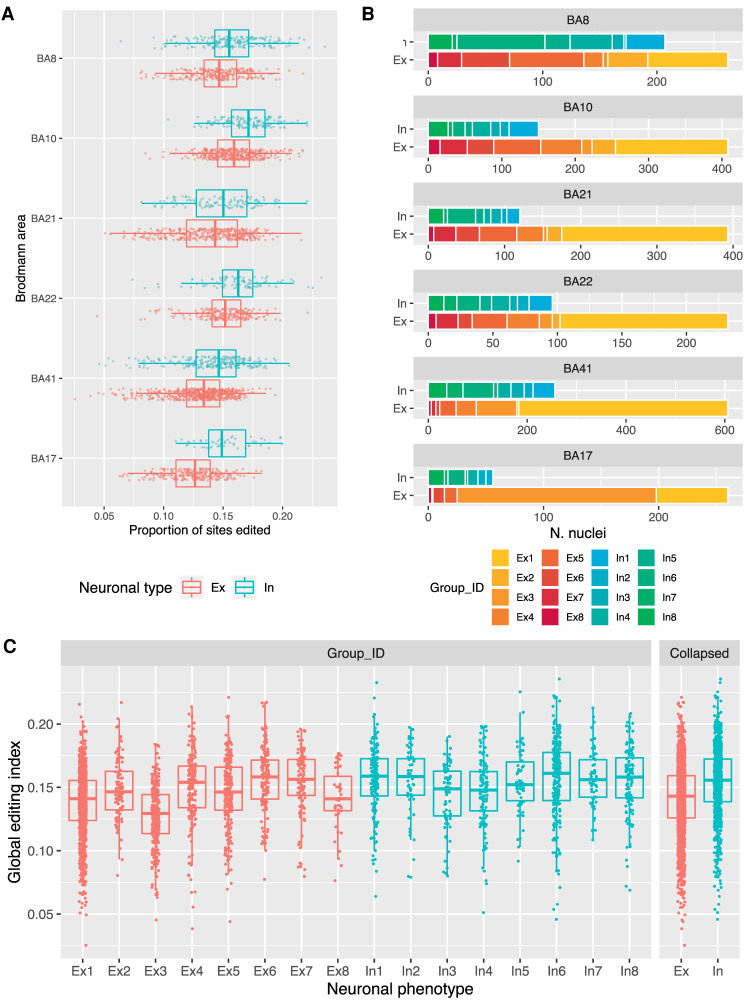
Editing differences between cortical regions and neuronal subtypes. (*A*) Distribution of global editing index (GEI; i.e., mean minor [G] allele frequency for sites in each cell) across six cortical Brodmann areas (BA), for inhibitory (teal) and excitatory (red) neurons. All groups except for Ex neurons in BA10 and In neurons in BA22 show significantly different GEI compared to In neurons of BA8. (*B*) Abundance of neuronal subgroups between cortical regions, as defined by Lake et al. (2016). Note different *x*-axis scales. (*C*) Global editing index in neuronal subgroups and when collapsed into gross neuronal phenotypes. All subgroups are significantly different to Ex1 except for Ex8 and In3. In: inhibitory neuron; Ex: excitatory neuron.

To compare editing rates between neuronal type and cortical region, we calculated a “global editing index” (GEI), taken as the mean minor allele (G) frequency across the sites transcribed in each nucleus (Supplemental Table S5). Although there was no significant difference in the number of reads mapped to grouped excitatory or inhibitory neurons, a modest effect of library size on GEI was detected, and controlled for in subsequent statistical testing. Inhibitory neurons displayed significantly greater editing than excitatory neurons (*P* < 1 × 10^−30^), which was largely attributable to greater editing in the In6, and lower editing in the Ex3 neuronal subgroups identified by Lake and colleagues (Fig. 3C). These trends remained when more conservative (i.e., less sensitive) editing metrics were used, based on Alu sites only (“Alu editing index”), or Alu sites transcribed in at least 100 cells (Supplemental Fig. S6). Differences in mean GEI between cortical regions were related to different proportions of neuronal subtypes (Fig. 3A,B). Specifically, the visual cortex (BA17) was enriched for neurons of subgroup Ex3, and showed both the lowest proportion of inhibitory neurons (18%), and the lowest mean GEI (see Supplemental Tables S3 and S5 for linear modeling results). Similarly, the superior temporal cortex (BA41) was enriched for group Ex1 neurons, and showed a lower mean GEI than the frontal cortex (BA8 and BA10) in which inhibitory neurons (In1, In6, and In8) were more abundant.

### Differential gene editing

**FIGURE 4.**
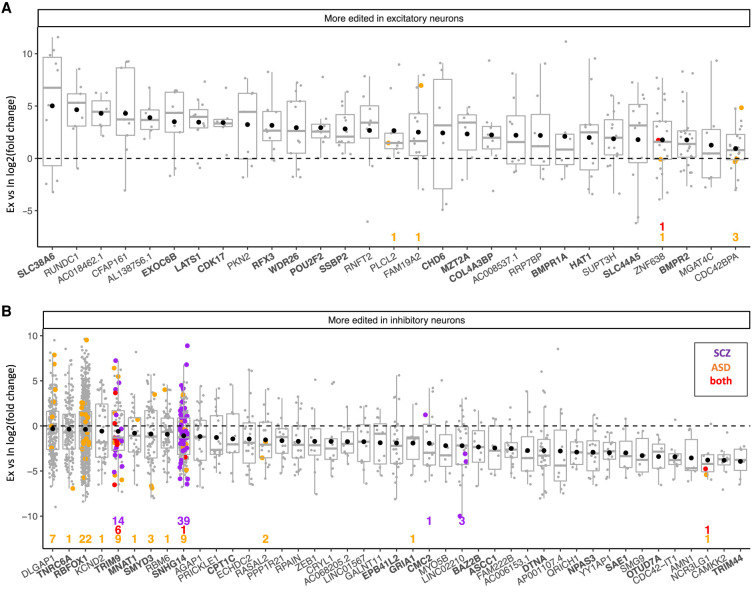
Differential gene editing between excitatory and inhibitory neurons. Genes with preferential editing in excitatory neurons (*A*) or inhibitory neurons (*B*). Each point represents log_2_ fold-change (*y*-axis) of an edited site in a gene (*x*-axis). Box plots indicate distribution of the middle 50% of editing fold-changes (box boundaries) and median fold-change (*middle* line). Black points represent the mean fold-change in editing per gene. Sites differentially edited in cohorts of individuals with schizophrenia (SCZ; purple) or autism spectrum disorder (ASD; orange) or both cohorts (red) are colored accordingly. The relevant number of such sites per gene is displayed numerically at the *bottom* of the panel. Labels for genes mentioned in the results section are displayed in bold.

To further interrogate RNA editing at the level of individual sites and genes, we aggregated single-cell editing signals into “pseudobulk” RNA samples and applied a differential editing framework which has been previously used to quantify differential DNA methylation (Chen et al. 2017). Briefly, this method uses multivariate generalized linear models to identify significant differences in edited (alternative) and unedited (reference) allele counts between grouped cell transcriptomes (see Materials and Methods). We compared editing between excitatory and inhibitory neurons by creating pseudobulk RNA samples (collapsing RNA-editing counts across cells occupying the 16 neuronal subgroups identified by Lake and colleagues), and correcting for cortical region of origin. Whereas no individual site was significantly differentially edited after correction for multiple testing, this framework permitted adaptation of gene set enrichment analysis (Wu et al. 2010) to identify individual genes enriched with editing sites. We found 72 genes that were enriched with edited nucleotides in one neuronal subtype (FDR < 0.1; Supplemental Table S6). Among 29 genes preferentially edited in excitatory neurons were the putative glutamate transporter gene *SLC38A6*, and the neuronal growth and migration factors *PKN2* and *FAM19A2*. Editing in transcriptional regulators was particularly notable, in *RFX3, POU2F2, CHD6, CDK17, SSBP2, HAT1*, and *SLC44A5* (Fig. 4A). Lastly, among transcripts preferentially edited in excitatory neurons were pro-survival proteins (*LATS1, BMPR1A, BMPR2*, and *WDR26*), cell cycle and division proteins (*MNAT1, MZT2A*), and nervous system development proteins (*EXOC6B, COL4A3BP*).

The remaining 43 dEd genes were more edited in inhibitory than excitatory neurons. Included in this group was *RBFOX1*, encoding a splicing regulator, and eight of its binding target genes (Weyn-Vanhentenryck et al. 2014). The small nucleolar host gene *SNHG14* was also preferentially edited in inhibitory neurons, as were transcriptional regulators and RNA-binding proteins, represented by *YY1AP1*, *ZEB1*, *NPAS3*, and the RNA-interference enzyme *TNRC6A*. Preferential editing of several post-translational modification enzymes was notable, including the sumoylation enzyme *SAE1*, histone methyltransferase *SMYD3*, and the de-/ubiquitylation enzymes *TRIM9*, *TRIM44*, *OTUD7A*, *CDC42*, and *BAZ2B*. Interestingly, editing at four of five candidate sites in the AMPA glutamate receptor subunit 1 (*GRIA1*), which oligomerizes with *GRIA2*, was more prevalent in inhibitory neurons. The excitatory neuronal marker gene *CDKK2* also showed a strong editing bias in these cells. Lastly, preferential editing in inhibitory neurons was identified in genes involved in mitochondrial metabolism: carnitine palmitoyltransferase 1C (*CPT1C*), and metallochaperone *CMC2*; as well as genes related to neurogenesis and synaptic function (*ASCC1*, *DTNA*, *DLGAP*, and *EPB41L2*).

A similar analysis of editing within each cortical region compared to the mean of the others, correcting for cell type, identified 99 dEd sites across 90 genes. Whereas no gene was significantly enriched with edited sites in any region, the majority of dEd sites were identified in the frontal cortex (BA8: 48 sites; BA10: 24 sites), with 14 dEd sites identified in the temporal cortex (BA22), and fewer than ten in other cortical areas. Three individually significant dEd sites were identified in *RBFOX1* and *RIMS2* respectively, with two sites each in *DNAJC11*, *EPN2*, *PEAK1*, and *TPST1* (Supplemental Table S7). Taken together these results suggest that major editing differences generally reflect neuronal phenotype rather than cortical region of origin.

### Intersection of differentially edited sites in neuropsychiatric illness with neuronal subtypes

Hundreds of RNA sites are reported as differentially edited in the brains of individuals with schizophrenia (Breen et al. 2019) and autism spectrum disorder (ASD) (Tran et al. 2019) relative to healthy controls. We tested whether genes that were dEd between inhibitory and excitatory neuronal nuclei were enriched with sites related to these conditions. Indeed, 71 sites dEd in ASD were localized within 12 genes that were more edited in inhibitory than excitatory neurons. In the same manner, 60 of 66 sites dEd in schizophrenia were concentrated in the *TRIM9* and *SNHG14* genes. Conversely, only seven dEd sites in ASD and one in schizophrenia were identified among genes displaying increased editing in excitatory neurons (Fig. 4A). Although dEd sites associated with these conditions were 2.6 times more likely to be present in genes dEd in inhibitory neurons, this was not significant by Fisher's exact test (*P* = 0.16).

### Small nucleolar RNA abundance is a marker of nuclear RNA editing

Abundance of ADAR family enzymes is at best a modest predictor of editing activity across human tissues (Roth et al. 2019), which has prompted a search for other modulators of editing (Tan et al. 2017; Quinones-Valdez et al. 2019). Prior to investigating correlations between editing and gene abundance, we confirmed that noncoding RNAs were fairly represented in the sNuc-seq data by comparing the rank abundance of the entire transcriptome with a human brain transcriptome generated using TGI reverse transcriptases (TGIRT), which produces minimally biased representations of relative transcript abundance (Nottingham et al. 2016). We found that sNuc-seq data correlated more strongly with the TGIRT Human Brain Reference than with TruSeq-based GTEx BA9 tissue, indicating accurate quantification of most gene biotypes (Supplemental Fig. S7). To identify potential novel modulators of editing, we then modeled GEI as a function of gene abundance, controlling for neuronal type. A total of 170 genes were significantly associated with GEI, with absolute effect sizes greater than 0.01 (FDR < 0.05) (Supplemental Table S8). Because these significant genes were among those most consistently expressed across cells (mean *n* cells per gene = 2250 and 687 for significant and nonsignificant genes, respectively), to allow a fairer comparison we down-sampled the data to a maximum of 1500 cells per gene. All results remained significant.

**FIGURE 5.**
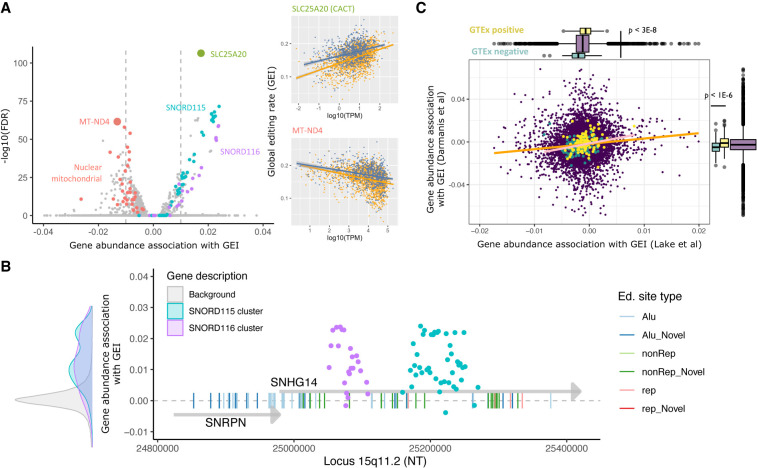
Associations between transcriptional abundance and global RNA editing rates. (*A*) Associations between gene abundance and the cell-wise global editing index (GEI) (*x*-axis). The transformed FDR-adjusted *P*-value of association is represented on the *y*-axis (higher values are more significant). Correlation plots at *right* display relationship between global editing index (*y*-axis) and log_10_ transcripts per million (*x*-axis) for neuronal nuclei expressing genes of interest: mitochondrial NADH oxidase 4, and mitochondrial carnitine transporter CACT. (*B*) Abundance of SNORD115 and -116 snoRNAs in locus 15q11 correlates with global RNA editing rates (*y*-axis). Genomic location is displayed in nucleotide units on the *x*-axis. The *y*-axis displays the effect size of transcriptional association with editing for SNRPN, SNHG14 (gray arrows), and SNORD cluster genes (points). The distribution of correlations between the SNORD clusters and editing is displayed at *left* relative to all other genes (background) and scaled to the *y*-axis. Edited sites in SNRPN and SNHG14 are represented by *vertical* lines, and colored according to genomic context. (*C*) Correlation between gene abundance-editing associations in ∼15K genes identified in two sNuc-seq data sets. Each purple point represents a gene. Associations between log abundance and global editing in the Lake et al. data set (*x*-axis) are displayed relative to results from Darmanis et al. data set (*y*-axis). The orange line indicates fitted correlation between data sets; the pink line indicates correlation between a subset of genes (yellow and green points) identified to have RNA editing associations in GTEx data. Marginal box plots indicate significant separation of positive (yellow) and negative (green) results from GTEx in both sNuc-seq data sets.

Consistent with previous reports (Tan et al. 2017; Roth et al. 2019), abundance of ADAR family genes was not associated with GEI at the single-nucleus level. In contrast, 47 small nucleolar RNA genes transcribed from the locus 15q11 showed positive correlations with GEI (Fig. 5A). This result was due to independent contributions from both neuronal subtypes, and was robust to the removal of a large cluster of edited sites in the snoRNA precursor transcript *SNHG14* (Fig. 5B). Another four snoRNAs from the *SNORD3* cluster on chromosome 17 were detected together with *SNORD114* (chromosome 14) and H/ACA snoRNAs transcribed from chromosomes 1, 3, and 20. Other noncoding RNAs including three small nuclear RNAs and eight ribosomal RNAs were also positively associated with GEI. Only two protein-coding transcripts in this group were identified: the mitochondrial carnitine transporter *CACT*, and the lipid transport protein *APOA1*. We curated the predicted interacting partners of editing-associated snoRNAs as detailed in Supplemental Methods. When collapsed across all nuclei, the mean editing rate of 35 snoRNA target genes was nonsignificantly higher than for nontarget genes (29% vs. 27%) (Supplemental Table S9).

Transcripts whose abundance was negatively associated with editing, related to *trans*-synaptic signaling (calmodulins 1 and 2, microtubule associated proteins *MAP1A* and *1B*, *SNAP25*, and syntaxin binding protein 1), and metallopeptidase protein-coding genes (*DPEP1*, *MMP10*, and *TRABD2B*)—some with roles in cellular differentiation (ADAM metallopeptidase TS20). Greater abundance of coagulation factor X (*F10*) and leucine-rich repeat protein 15 (*LRRC15*) was also negatively associated with GEI, as was the abundance of seven long noncoding RNAs. Also in this group were the Alu repeat-containing transcripts *7SL* and olfactory receptor *OR52A5*, and genes related to neurogenesis (chimerin 1 and olfactomedin). Transcripts encoding proteins with transcription factor activity (*PAX6*, *IRX2*) and several related to neurotransmission (*LYPD4*, tryptophan 5-monooxygenase, the active calcium transporter *ATPB2* and potassium channel protein *KCNJ15*), were also negatively associated with GEI. Lastly, RNA levels of heat shock protein 90 and mitochondrial NADH oxidase showed a negative relationship with editing. When the effect-size threshold was omitted, we observed enrichment of nuclear-encoded mitochondrial genes among the negative transcriptional correlates of editing (Fig. 5A).

To test the consistency of these findings across adult neurons, we performed the same analysis on the 116 sNuc-seq neuronal transcriptomes assayed by Darmanis et al. (2015). Whereas ADAR enzymes were again not significantly associated with editing, the calmodulins 1 and 2, SNAP25, beta actin, MAP1B and protein phosphatase 3CA transcripts showed negative associations in both data sets. There were no common genes positively associated with editing between the two data sets, which may be due in part to significant differences in the classes of assayed RNA biotypes. Nevertheless, the correlation between editing associations (beta values) was positive and significant (*R* = 0.11, *P* < 1 × 10^−30^; Fig. 5C). Furthermore, genes with positive editing associations in bulk RNA-seq from human tissues (Tan et al. 2017) showed similar positive trends in results from both single-nucleus data sets. Conversely, genes with negative editing associations in GTEx tissues showed congruent negative associations in single-cell data (*R* = 0.26, *P* < 1 × 10^−4^), and were significantly segregated from positively associated genes (Fig. 5C, marginal boxplots). Taken together, these results suggest extensive involvement of small nucleolar and other noncoding RNAs in RNA editing, and a spectrum of transcriptional associations with editing that is broadly conserved in single neurons.

## DISCUSSION

RNA editing is ubiquitous in the human central nervous system, and aberrant editing is implicated in numerous neurological/neuropsychiatric diseases, cancer, and immune disorders. Here we undertook the first high-resolution exploratory investigation of this process in single neuronal nuclei assayed across six cortical regions from a neurotypical adult brain donor, and found extensive agreement with previously reported edited sites in healthy and disease-affected brain tissue. Further, given that the majority of existing single-cell brain studies contain poly(A)-targeted data generated on the 10× platform, the abundance of nuclear/nucleolar pre-mRNA species, and ncRNA species in this data set profiled with full-length capture technology, remains a rare and valuable resource. Our analysis of sNuc-seq data therefore facilitated deep insight into editing activity across the nuclear transcriptome occurring in physical proximity to brain-specific ADAR enzymes (Gallo et al. 2017). By applying a new, sensitive linear modeling approach to detect differential site- and gene editing across cell types and cortical regions, we demonstrated that RNA editing analysis is feasible in sNuc-seq data, and informative for investigating disease-relevant sites in the context of specific neuronal cell types. We further identified editing differences in disease-related genes between neuronal subtypes, and a strong association between snoRNA abundance and transcriptome-wide RNA editing.

This work was made possible by publicly available sequencing data and curated databases. The novel sites identified here require further validation and functional studies, and will constitute the first publicly available cell-type specific editing reference database. The main limitations of this study lie in the lack of RNA stranding information in the sNuc-seq data, and the lack of reference genomes for brain donors—both of which hamper our ability to disambiguate editing sites from genomic SNPs. To address these concerns, we took stringent measures including: (i) removing common genomic SNPs from the results, (ii) removing sites derived from transcripts that overlap on opposing strands; and retaining only those sites (iii) detected within genes on the cognate strand (iv) covered by at least five reads, and (vi) detected in at least ten nuclei. The latter filter should eliminate PCR and other random artifacts because SMART-seq library preparation is independent for each nucleus. We note that these thresholds are quite conservative, and therefore detection of the RNA editing signal is likely not saturated in the filtered data set, meaning further independent investigations in this type of data will likely further increase the catalog of known editing sites.

The proportionate distribution of edited sites across RNA motifs (Alu and non-Alu repeats) and genic features (mostly intronic or 3′-UTR) broadly agrees with the original characterization of editing in the human transcriptome (Li et al. 2009b). Excitingly, thousands of putative novel editing sites were detectable in the present data, thanks to the extensive coverage of the transcriptome at single-cell resolution. That a substantial proportion (58%) of 9285 newly identified sites occurred within canonical Alu/repetitive non-Alu contexts, and that 26% were detectable in independent sequencing of neurons from unrelated individuals, suggests that this data type contains novel and reproducible insights which are not detectable in bulk RNA sequencing. Indeed, the cellular heterogeneity of RNA editing is a striking result in the present study.

Whereas previous studies of bulk RNA portray editing as a pervasive low-frequency phenomenon (Tan et al. 2017), our results suggest that this is, at least in part, an artefact of high frequency editing activity that is restricted to subsets of cells, and thus masked by ensemble averaging (Supplemental Fig. S4). Accordingly, a fundamental reevaluation of our understanding of this process is warranted as more sNuc-seq data at higher coverage becomes publicly available. Routine integration of editing and expression data in single-cell studies has great potential to inform the structural and functional heterogeneity of the brain. As the present data is enriched for nuclear RNA, it likely predominantly reflects editing activity of ADAR2, which is spatially restricted to the nucleus and predominantly expressed in neurons. Conversely, highly prevalent but low-penetrance editing, and sites edited in cytosolic RNA, will be underrepresented in the current data.

### Site-specific editing

Our study was able to survey editing sites across cortical regions and neuronal subtypes with high resolution, as well as investigate the transcriptomic underpinnings of neurological and psychiatric illness in the aggregated data, and in the context of cell type and cortical region. Most editing in neuronal nuclear pre-mRNA occurs in intronic regions where the inference of functional effects is difficult. It was therefore surprising to find enrichment of ASD and epilepsy disease associations among the pre-mRNAs that were predicted to undergo multiple structural conformation changes upon editing. In noncoding RNA, this process modulates intronic retention, nuclear export and protein translation rates, all of which likely depend on the steric accessibility of protein-binding motifs. Although these heavily edited disease gene sets act in many different biological pathways, this finding suggests that susceptibility to editing-related structural changes may unify neuropsychiatric disease genes. We hypothesize that subtle editing-related structural effects on multiple transcripts may mediate the pervasive psychological effects associated with ASD and epilepsy, which often co-occur. We also report a relative abundance of genes with low amino acid substitution tolerance among the putative structurally affected transcripts. Together these findings appear to support constraints on both the coding sequence and allowable RNA structural conformations of these uniquely important genes.

Conversely, we found that a number of transcripts encoding putative editing-dependent missense sites, are involved in adenosine binding. Editing-based feedback loops in editing-related enzymes are reported in model systems (Feng et al. 2006), and may be more extensive than previously thought. Beyond adenosine binding proteins, the presence of putative recoding sites in synaptic transmission-related proteins, as well as solute carrier family members and neurotransmitter receptors, suggests that editing-based peptide modification modulates both the sensitivity of neurons to stimuli, and the responsivity at the synapse.

### Differential editing between cell types and cortical regions

Comparison of the global editing index (“GEI”) between cell types and cortical regions was facilitated by averaging the editing frequencies of transcribed sites in each cell. In the frontal cortex, both major cell types exhibited a higher GEI than other regions. Interestingly, Brodmann areas 17 and 41 house the primary visual and auditory cortices, respectively, and exhibited lower global editing rates. If higher editing rates are assumed to dampen neuronal activity in both inhibitory and excitatory cell types, then these regional differences may support the prevailing understanding of the functional organization of the cortex, wherein sensory regions provide strong excitatory input (corresponding to reduced editing); and the frontal cortex is primarily inhibitory, acting to increase the salience of selected perceptual inputs (Hu et al. 2013).

Using a pseudobulk approach to assess differential editing revealed that editing differences between cortical regions manifested in differentially edited sites alone, whereas differences between cell-types were significant in aggregate at the gene level, but not at the site level. It may be that the volume, rather than identity of edited sites across transcripts distinguishes different cell types. Differentially edited genes in excitatory neurons tended to contain relatively small numbers of edited sites showing large fold-changes. This is consistent with the lower global editing rates observed in excitatory cells. One of several novel findings is the prominent editing of a putative glutamate transporter, *SLC38A6*, which is notable given the high requirement in these cells for glutamate—the primary excitatory neurotransmitter. Nearly a quarter of genes preferentially edited in excitatory neurons encode transcription factors or histone modification enzymes, pointing to potential feedback between editing and transcription. In addition, heightened editing in excitatory neurons of transcripts involved in self-renewal of neural progenitors (*LATS1*) and prosurvival proteins (*WDR26*, *BMPRs 1A* and *2*), suggests that a primary role of editing in these cells could be to contribute a homeostatic signal. This is supported by negative associations between editing and abundance of both nuclear mitochondrial genes, and four cell-type markers of the Ex1 subgroup. It may be that prosurvival transcripts act as sensors of excessive mitochondrial respiration, which impinges on RNA editing and viability in excitatory neurons. We stress however that the negative association between mitochondrial gene abundance and editing is also seen in bulk tissue sequencing (Tan et al. 2017), and is unlikely to reflect apoptosis in this case, as any nuclear libraries containing more than 15% mitochondrial RNA were removed during preprocessing. Complex interactions between mitochondrial metabolism and editing are also suggested by the strong positive association between editing and abundance of the carnitine shuttle protein, *CACT*, required for fatty acid oxidation. Dysregulated editing of carnitine pathway genes was recently reported in ALS (Moore et al. 2019), and now warrants further molecular investigation.

Several transcripts assayed in inhibitory neurons exhibited high-density editing, namely *RBFOX1*, *TNRC6A*, *DLGAP1*, *TRIM9*, and *SNHG14*. These genes also encompassed vastly more sites reported as differentially edited in neuropsychiatric patient cohorts, most notably ASD. Sites linked to schizophrenia were largely restricted to the ubiquitin E3 ligase *TRIM9*, and *SNHG14*. The former protein may promote editing in inhibitory neurons by dampening the function of *PAX6*, whose abundance is negatively associated with editing in our data. Indeed, crosstalk between post-transcriptional and post-translational modification systems in these cells is also suggested by editing in transcripts central to synaptic formation including de/ubiquitylation enzymes (*TRIM* family and *OTUD7A*), *DLGAP1* and *DTNA*.

Increased editing of the ASD “hub” gene *RBFOX1* (Voineagu et al. 2011), its target genes (Weyn-Vanhentenryck et al. 2014), and other genes implicated in ASD (Parikshak et al. 2013), aligns with previous work implicating dysfunction of inhibitory interneurons specifically in this disorder (Lunden et al. 2019). RBFOX family genes are implicated in excising C/D box small nucleolar RNAs from their host gene *SNHG14* (Coulson et al. 2018), which was also heavily edited in inhibitory neurons. Genomic deletion of the SNORD115 and/or 116 cluster has been independently associated with Prader–Willi syndrome (Sahoo et al. 2008; Bieth et al. 2015), which features autistic traits (Descheemaeker et al. 2006). Although *SNORD115* RNAs are reported to direct editing and splicing of *5HT2C* serotonin receptors in vitro (Bratkovič et al. 2018), here we identified positive associations between abundance of *SNORD115*/*116* RNAs and editing across the entire transcriptome, which has not been observed previously. This association may have been revealed due to the relative enrichment of nucleolar RNA in this data set. Small RNAs such as SNORDs are rather poorly represented in the Darmanis et al. (2015) sNuc-seq data, and especially so in poly(A) enriched Tru-seq bulk brain RNA sequencing.

We hypothesize that locus 15q11 snoRNAs may recruit ADAR enzymes or stabilize their interactions with substrates in the nucleolus. Alternatively, these molecules may compete with ADARs for RNA substrates to fine-tune editing. Indeed, the large proportion of genes whose abundance is negatively correlated with editing in this study, and findings associating many more RNA binding proteins with negative rather than positive effects on editing (Quinones-Valdez et al. 2019), suggest that this epi-transcriptomic process may be constitutively active and subject to negative regulation. Together these findings indicate that the 15q11 region is a nexus of RNA editing-related activity whose transcription may be a proxy for global RNA editing rates. The extensive differential editing reported in 15q11 in this study between neuronal subtypes and cortical regions, and in two prominent neuropsychiatric disorders, makes this region a compelling subject for further investigation. It is also intriguing to consider the potential involvement of RNA editing dysregulation in neurodevelopmental conditions associated with structural variation in this locus (Ulfarsson et al. 2017; Jønch et al. 2019).

Although the effect size of transcriptional associations with editing were modest, they were similar in scale to those reported in analyses of bulk RNA which produced novel experimentally validated modulators of editing (Tan et al. 2017). Further, the overall agreement in the sign and magnitude of transcription-editing associations across sequencing technologies in independent studies, suggests that a complex network of interacting partners including noncoding RNAs, may affect RNA editing, and await experimental validation.

In conclusion, we integrated RNA editing signals in individual neurons with known editing sites in the healthy and diseased brain. Thousands of newly reported editing sites share broad characteristics of documented sites and replicate in independent single-cell data, but are likely subsumed by ensemble averaging in bulk tissues. Sensitive detection of differential site- and gene-editing revealed concentration of ASD-related gene editing in inhibitory neurons. Expression of the ASD-associated Prader–Willi locus snoRNAs mark global editing activity, and associations between editing and transcription are conserved across studies. This work adds exciting new dimensions to our understanding of post-transcriptional regulation in healthy adult cortical neurons, and a comprehensive reference for forthcoming investigation of editing in single-cell samples from neuropsychiatric patient cohorts.

## MATERIALS AND METHODS

### RNA sequencing data and reference databases

Raw data from full-length (SMART-seq) sequencing of single brain cell nuclei were downloaded from dbGaP (study accession phs000834; project ID 20576) (Lake et al. 2016). The neuronal subtype annotations assigned by the study authors based on gene expression profiles (eight excitatory and eight inhibitory neuronal subgroups) were also downloaded. Single nucleus SMART-seq data for 116 neurons from the anterior temporal lobe of four adult donors (GSE67835) (Darmanis et al. 2015) were downloaded from the Gene Expression Omnibus using the NCBI SRA toolkit. Metadata including the cell type and cortical area of cell origin were accessed from the sequence read archive and/or relevant Supplemental Material (Supplemental Tables S1 and S2). Raw counts for the universal human reference RNA and human brain reference RNA data sets assayed using TGIRT technology (SRP066009) (Nottingham et al. 2016) were downloaded via the recount2 server (Collado-Torres et al. 2017) and converted to transcripts per million for correlation with equivalent Lake et al. values. The REDIportal database (Picardi et al. 2017a), which incorporates the RADAR (Ramaswami and Li 2013) and GTEx human tissue RNA editing databases (Tan et al. 2017), was downloaded from http://srv00.recas.ba.infn.it/atlas/download.html. Additional reference databases were accessed as described in Supplemental Methods.

### RNA sequence processing and gene abundance quantification

RNA sequencing reads were mapped to the human reference genome (GRCh38.91) using the STAR aligner (Dobin et al. 2012) in two-pass mode with the splice junction database overhang maximized for the respective read lengths (47 for phs000834; 74 for GSE67835). Gene abundance was quantified using featureCounts with fractional assignment of multimapping reads (Liao et al. 2014). Single-nucleus SMART-seq data sets were further filtered to remove samples with abundant mitochondrially encoded RNAs (likely indicating apoptosis), low library complexity and low coverage. Count libraries were normalized for size and complexity, and clustered using scran (Lun et al. 2016) and scater (McCarthy et al. 2017). For cluster analysis of the phs000834 data, nuclei were colored according to the neuronal phenotype and cortical region designated by Lake et al. (2016).

### RNA editing detection and filtering

We called single nucleotide polymorphisms (SNPs) in the nuclear transcriptomes using the GATK best-practices pipeline (Van der Auwera et al. 2013) for RNA-seq data. This involved read deduplication, splitting, base quality score recalibration, and variant calling with HaplotypeCaller. No call confidence filter was applied at this step in order to retain all potential variants. Variant call format files were converted to GDS format using the R SeqArray package (Zheng et al. 2017), and combined with common genomic SNPs, and REDIportal sites. For phs000834, A > G and T > C sites covered by at least five reads, with a minor allele count of at least two, and detected in at least 10 high-quality (i.e., nonapoptotic, high transcriptional complexity) libraries, were retained. Common genomic A > G and T > C SNPs were extracted for use downstream. As SMART-seq data is unstranded, categorically determining the originally edited strand is not possible. Therefore, to enrich the data set for true editing sites, after removing common genomic SNPs, we imposed further filtering as follows. Sites were only retained if they were: (i) present in a previously published RNA editing database, and/or (ii) located within nonoverlapping regions of an ensemble feature, and on the cognate strand (i.e., A > G sites within a feature on the 5′ strand; T > C on the 3′ strand). The bedtools intersect module was used to locate previously unreported sites within genomic repeats (RepeatMasker); and to identify the genic feature (e.g., start codon, exon, intron, 3′-UTR etc.) occupied by sites within protein-coding genes (Quinlan and Hall 2010). Sites with insufficient coverage were distinguished from transcribed, un-edited sites using the samtools depth module (Li et al. 2009a). The Variant Effect Predictor (McLaren et al. 2016) was used to predict the molecular consequences of RNA editing sites. Only predicted effects other than “up/down-stream gene variants,” “intergenic” and “intronic variants” were reported. For GSE67835 the same site detection and filtering procedure was followed except a lower prevalence threshold (at least three nuclei) was applied given the smaller number of neurons under consideration.

### Statistical analysis

To assess the replication of editing sites detected in this and other studies, candidate sites that survived filtering were intersected with sites reported in bulk RNA sequencing data sets and single neuronal nuclei (Darmanis et al. 2015) (GSE67835) detailed above.

#### Editing site density

To characterize editing in Alu, repetitive non-Alu, and nonrepetitive contexts across nuclei, novel and previously reported sites were extracted. For each context category, the distance from each edited site to the nearest neighbor (regardless of nucleus ID) was calculated and displayed as a density function.

#### Disease enrichment

Edited sites were grouped by sequence context, and genes in which at least three sites of a single context were detected, were interrogated for disease associations using the R disease ontology enrichment analysis package DOSE (Yu et al. 2015). The set of genes expressed in at least 300 neuronal nuclei (i.e., >10% of total) was supplied as the background (minimum and maximum gene set sizes of five and 1000, respectively). Disease ontologies supported by at least two edited genes were considered for further analysis. Those ontologies with an enrichment *Q*-value <0.1 were considered significant.

#### Predicted RNA structure perturbation

The effect of A > I (G) editing on local RNA structure for nonoverlapping sites was estimated using RNAsnp software (Sabarinathan et al. 2013) based on a region of 441 nt of RNA flanking upstream (220 NT) and downstream (220NT) of each edited site. Genomic RNA sequence was extracted using the Bioconductor package BSgenome.Hsapiens.UCSC.hg38 (Team TBD 2020), and flanking regions were trimmed in cases where the edited site was proximal to a gene boundary. In accordance with empirical *P*-value distributions calculated by Sabarinathan et al. (2013), results with *P*-values <0.1 were considered significant.

#### Differential editing

Differential editing was assessed at the level of whole nuclei, aggregated over all transcribed candidate editing sites; and between genes, using a pseudobulk framework. A nucleus-level “global editing index” (GEI) was quantified as the mean minor allele (G) frequency for candidate editing sites in each cell. This metric was compared with an “Alu editing index” (AEI), composed of Alu sites only; and a more stringent AEI comprising Alu sites transcribed in at least 100 cells. Edited sites were subsequently grouped by transcriptomic context (Alu repeats, repetitive non-Alu sequence, and nonrepetitive sequence) to determine the contribution of each to global editing.

Given the sparse coverage in single-nucleus RNA sequencing, in order to robustly detect differential gene-level editing between neuronal subtypes controlling for cortical region, we adapted a counts-based framework to test for differential editing. Specifically, pseudoreplicates were created by summing counts of reference (unedited) and alternate (edited) alleles at sites with count variances >0.2, across neurons in phenotypically similar subgroups as defined by Lake et al. (2016) (Supplemental Table S1). This allowed testing differential site-wise editing between gross neuronal subtypes while controlling for cortical region, using a negative binomial model of gene expression in the limma R package, as has been successfully applied for differential methylation analysis (Ritchie et al. 2015; Chen et al. 2017). In contrast to ratiometric testing approaches, this method takes site coverage into account, thereby minimizing the probability that lowly expressed, highly variable sites are called as significant. Differential editing at the whole-gene level was identified by testing for enrichment of sites with consistent directional fold changes in editing between neuronal subtypes or cortical regions, using the fry rotation testing module in the limma R package (Wu et al. 2010).

Relationships between length-normalized log-transformed neuronal transcript abundance (transcripts per million) and global editing index were assessed with linear models, controlling for gross neuronal phenotype (excitatory or inhibitory) and library size unless otherwise stated. Gene set Gene Ontology enrichment analysis was performed on transcripts significantly associated with editing using the limma goana module (Ritchie et al. 2015), after exclusion of genes with absolute effect size estimates less than 0.01.

Data transformation and analysis was performed in R (R Core Team 2014), using the dplyr (Wickham et al. 2015), tidyr (Wickham 2021), and broom (Robinson et al. 2021) packages. doParallel (Weston and Microsoft Corporation 2020) was used for parallel processing. Data were visualized using ggplot2 (Wickham 2016), ggridges (Wilke 2021), ggforce (Pedersen 2020), ggExtra (Attali and Baker 2019), ggpubr (Kassambara 2020), cowplot (Wilke 2019), and UpSetR (Conway et al. 2017) packages.

## SUPPLEMENTAL MATERIAL

Supplemental material is available for this article.

## Supplementary Material

Supplemental Material
