# Supplementary material for: A survey of RNA editing at single-cell resolution links interneurons to schizophrenia and autism
Source: RNA. 2021 Dec;27(12):1482–96. doi: 10.1261/rna.078804.121 (PMC8594476; doi:10.1261/rna.078804.121)
Supplement: Supplemental Material [file supp_27_12_1482__DC1.html]

A survey of RNA editing at single cell resolution links interneurons to schizophrenia and autism — A survey of RNA editing at single cell resolution links interneurons to schizophrenia and autism — A survey of RNA editing at single-cell resolution links interneurons to schizophrenia and autism — Supplemental Material 

# A survey of RNA editing at single-cell resolution links interneurons to schizophrenia and autism

## Supplemental Material

- Supplementary\_Figures.pdf
- Supplementary\_Methods.docx
- Supplementary\_Tables.xlsx
