# Supplementary material for: A survey of RNA editing at single-cell resolution links interneurons to schizophrenia and autism
Source: RNA. 2021 Dec;27(12):1482–96. doi: 10.1261/rna.078804.121 (PMC8594476; doi:10.1261/rna.078804.121)
Supplement: Supplemental Material [file supp_078804.121_Supplementary_Figures.pdf]

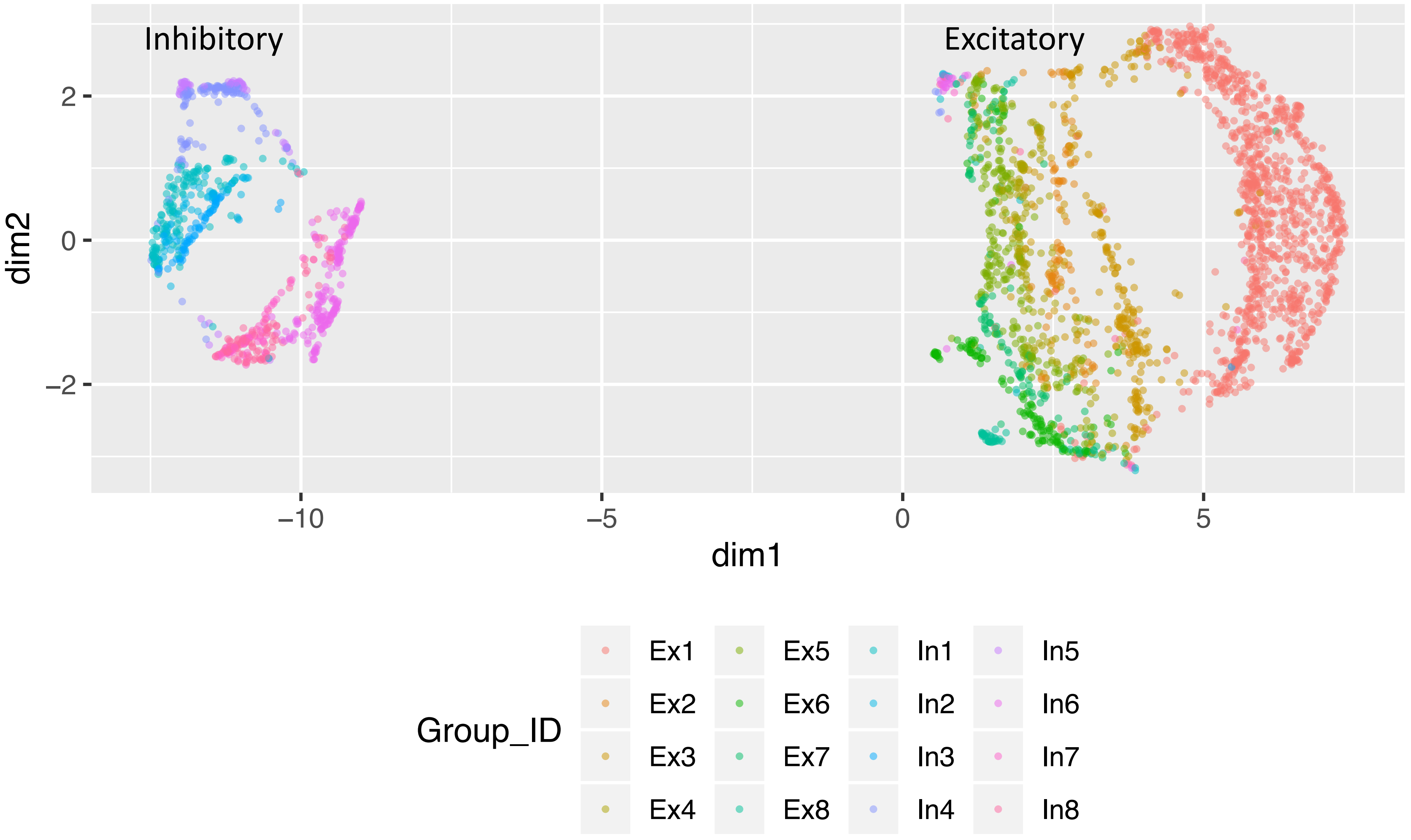

SFigure 1. Independent clustering of 3055 high-quality neuronal nuclear transcriptomes separates inhibitory from excitatory neurons. UMAP plot displaying independent clustering of neuronal transcriptomes based on gene abundance. Each point represents a neuronal nuclear transcriptome, coloured by cell type as assigned by Lake and colleagues (2016). Inhibitory neurons (In) area at left, and excitatory neurons (Ex) at right. Cells with low library sizes or containing >15% mitochondrial RNA were excluded during quality control.

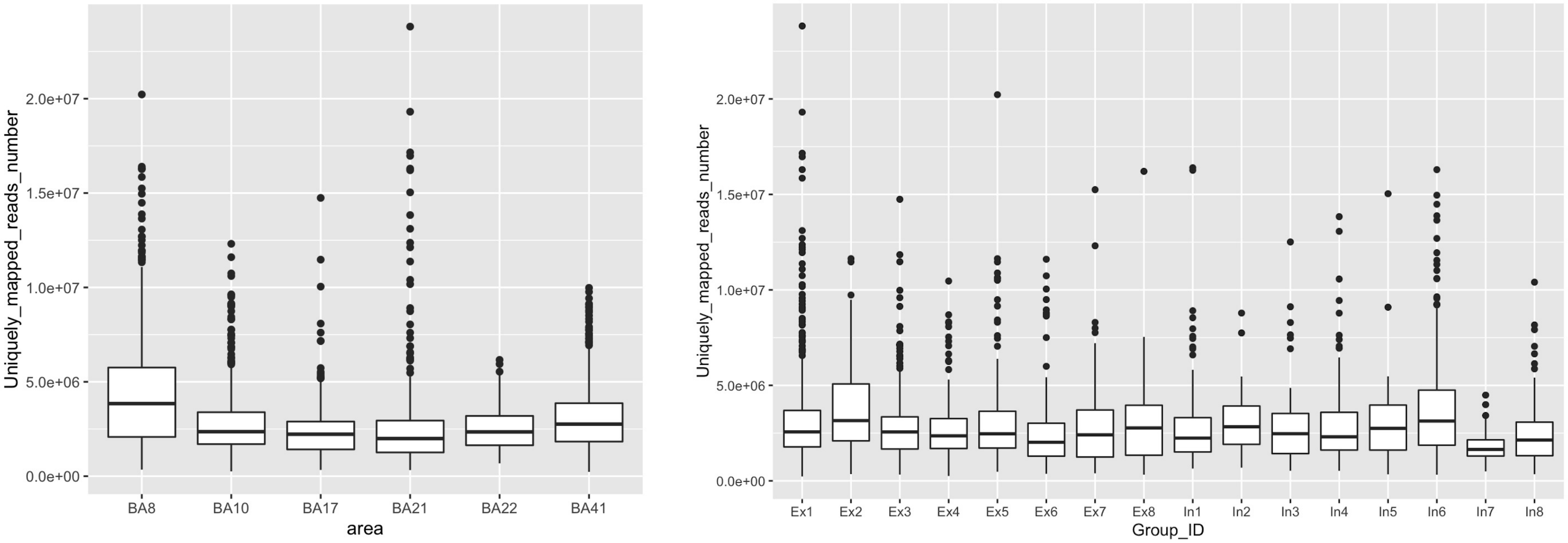

SFigure 2. Read mapping statistics for neuronal nuclear transcriptomes, grouped by cortical area, and neuronal sub-group. The average number of uniquely mapped reads was 3 million. The mean number of multi-mapping reads was 780,535. When quantifying gene abundance, a fractional assignment method was used for multi-mapping reads (see methods). BA8 showed significantly more uniquely mapped reads than other cortical regions; and neuronal sub-groups In7 and In8 had significantly lower numbers of uniquely mapped reads; whereas Ex2 and In6 had higher numbers.

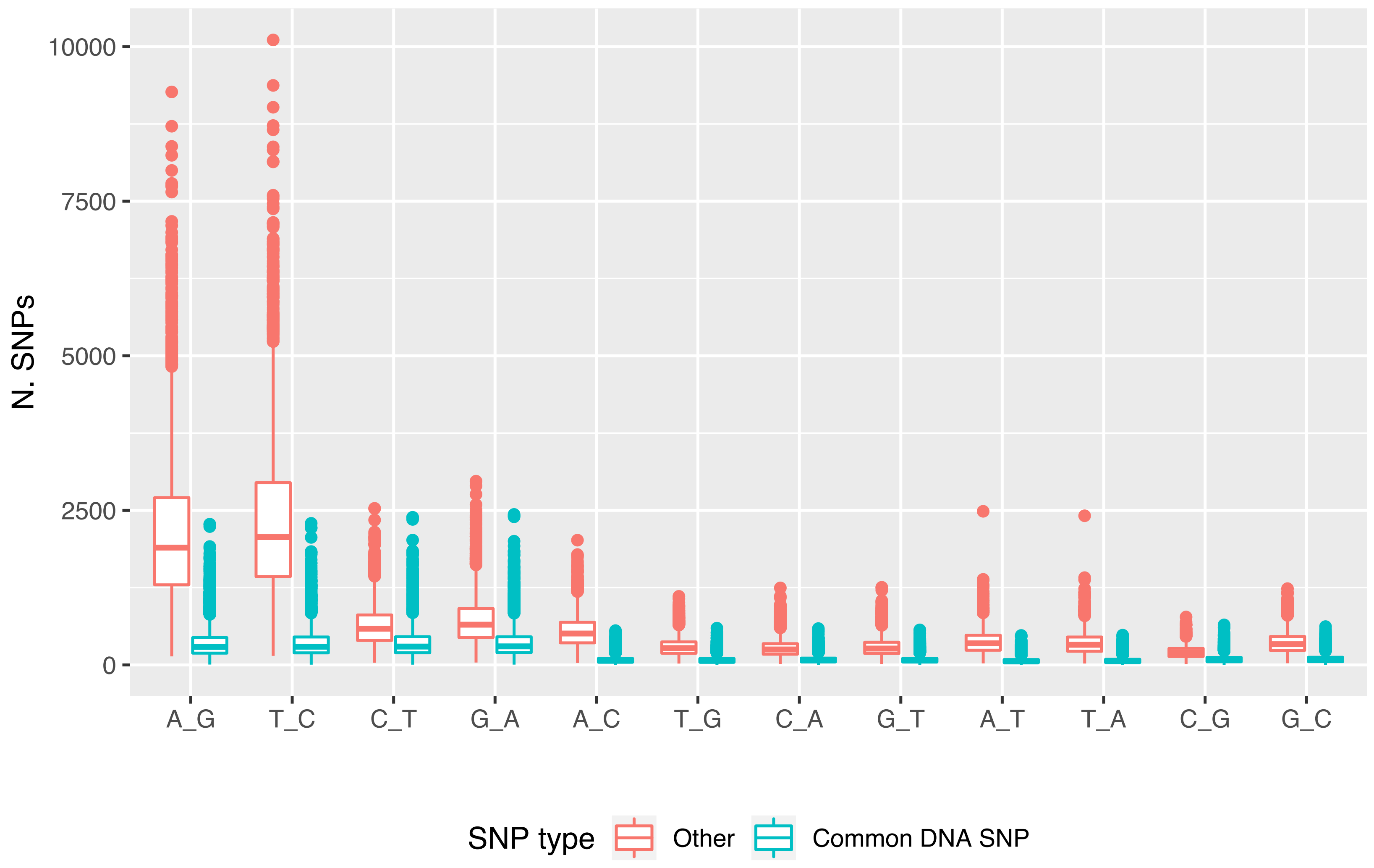

SFigure 3. RNA editing signal is prominent in raw SNP call data. Distribution of number of SNPs (y axis) detected per nucleus in raw SNP-call data, separated by SNP type. SNP substitutions (DNA reference_RNA nucleotide) are indicated on x axis. 'Other' (red) SNPs encompass a strong RNA editing signal (A>G for forward DNA strand; and T>C for reverse DNA strand), distinguished from sites catalogued in the common human SNP database, dbSNP (green).

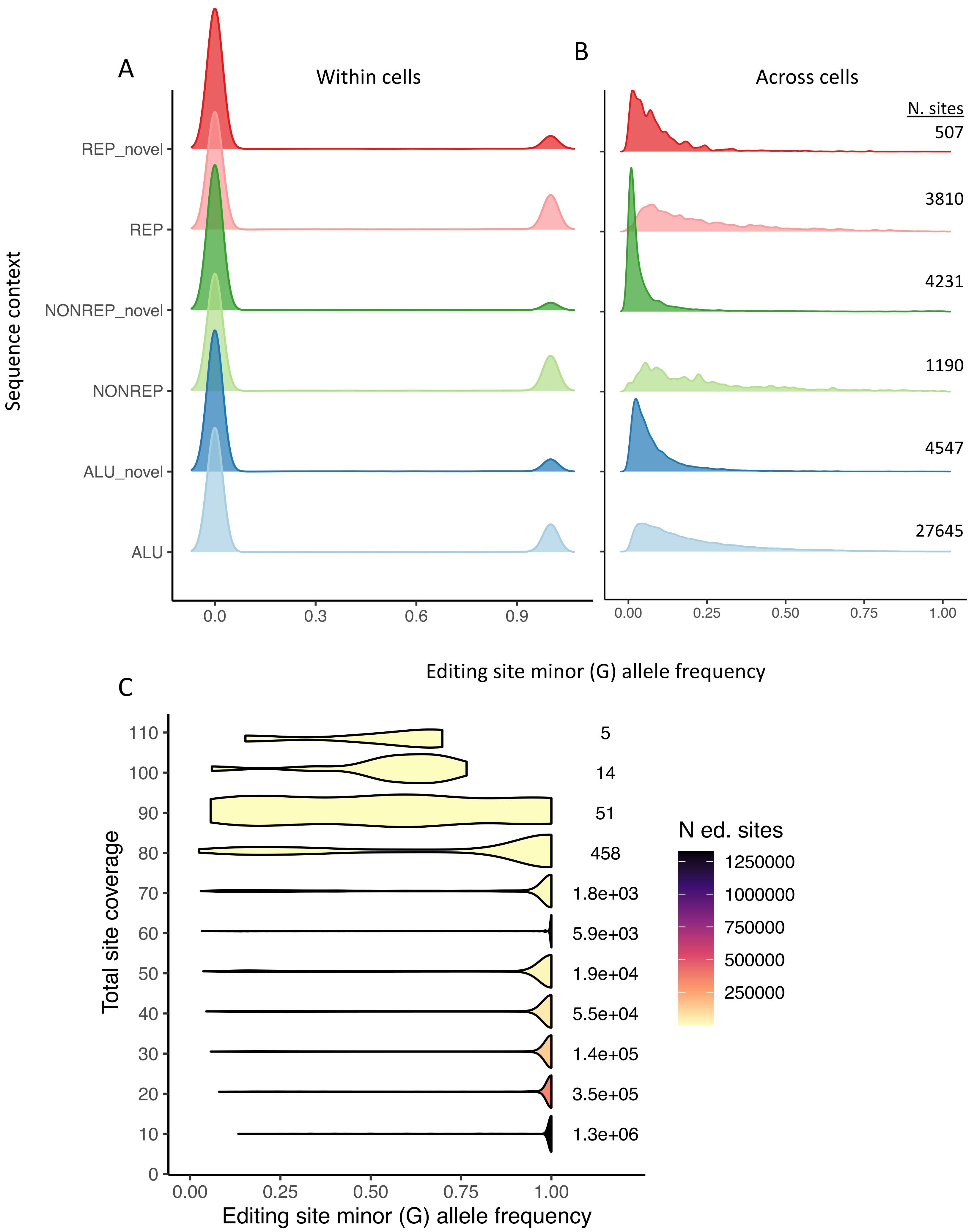

SFigure 4. Edited (G) allele frequency distribution (x axis; all panels) within and across cells. A) Density of editing allele frequency within cells. The sequence context for edited sites is displayed on the y axis. B) Density of editing allele frequency per site across cells. The number of sites identified in each sequence context for panels A and B are displayed at right. At the single nucleus level, candidate sites are generally completely edited, or unedited. When averaged across nuclei, the minor allele frequency follows the positive skew reported in analyses of bulk RNA editing. C) Edited allele frequency for all ~1.9m editing events in the dataset. The mean edited allele frequency is close to 1 for sites with total read coverage ranging from 10 to 70 reads, encompassing the vast majority of detected editing events. This suggests that the bimodal distribution in panel A is not an artefact of low read coverage.

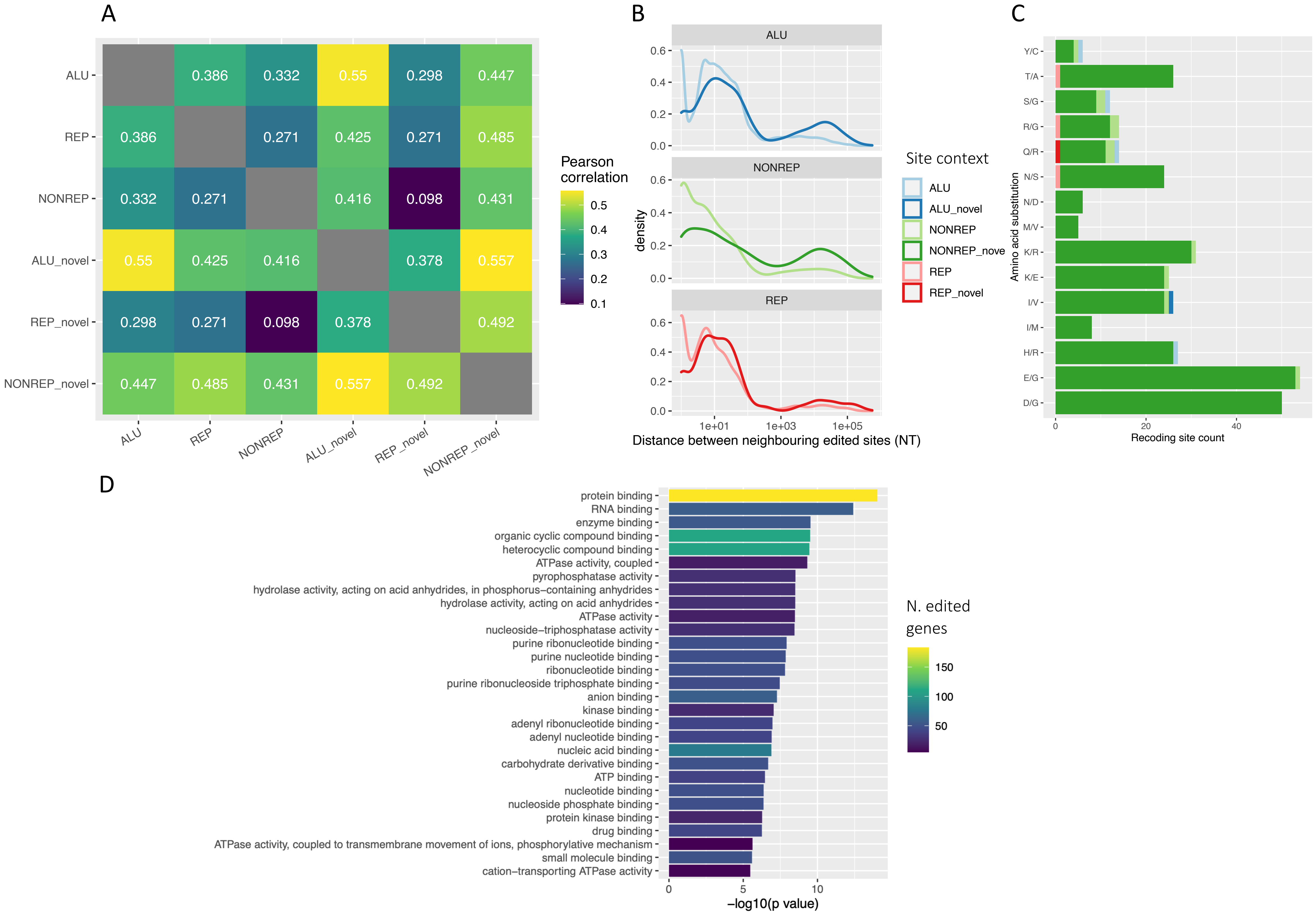

SFigure 5. A) Correlations between log10 (number of sites per gene) for different editing sequence contexts (x and y axes). Numbers in tiles represent Pearson correlation values. Novel sites are those not previously catalogued in REDIportal, RADAR or GTEx databases, which are defined based on intersection with the Repeat Masker database (containing Alu and non-Alu repetitive regions). B) Density distributions for distance to nearest-neighbour, coloured by site context, collapsing across nuclei. Alu and repetitive non-Alu sites occur in clusters with a peak of separation around 10 NT, whereas non-repetitive sites are more distantly interspersed. C) Predicted protein re-coding consequences of 233 exonic sites in 170 genes. Number of sites (x axis) encoding different amino acid substitution consequences (y axis), coloured by editing sequence context. D) Top 20 most enriched molecular function Gene Ontology terms (y axis) among 232 putative re-coding target genes. Bar length indicates enrichment significance (x axis) and fill colour indicates the number of edited genes supporting each result.

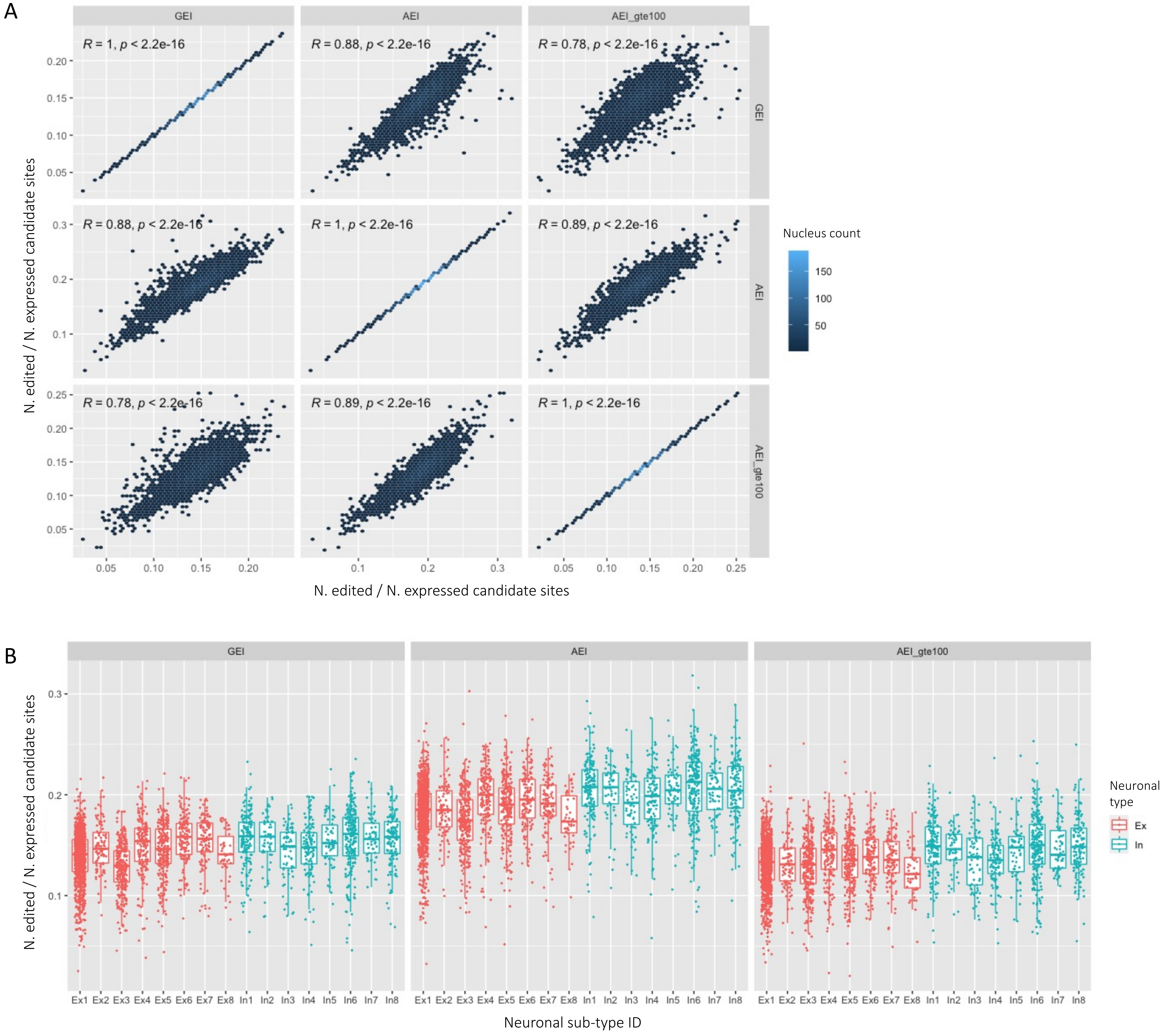

SFigure 6. A) Correlations between cell-wise editing rates calculated using different sets of 41,930 high-confidence editing sites. GEI: global editing index, calculated using all transcribed candidate sites per cell; AEI: Alu editing index, calculated using all transcribed Alu sites per cell; AEI_gte100: stringent AEI, calculated using only Alu sites transcribed in at least 100 neuronal nuclei. Hexagons indicate frequency bins, coloured by number of observations per bin (‘count’). Pearson’s product-moment (R) and p value are displayed at top. B) Cell-wise editing rates (y axis) for neuronal sub-groups (x axis) calculated using the global editing index (GEI; as for Figure 3c); the Alu editing index (AEI); and the stringent AEI (AEI_gte100). Inhibitory neurons (In) show significantly greater editing than excitatory neurons (Ex) as a group irrespective of the editing index used.

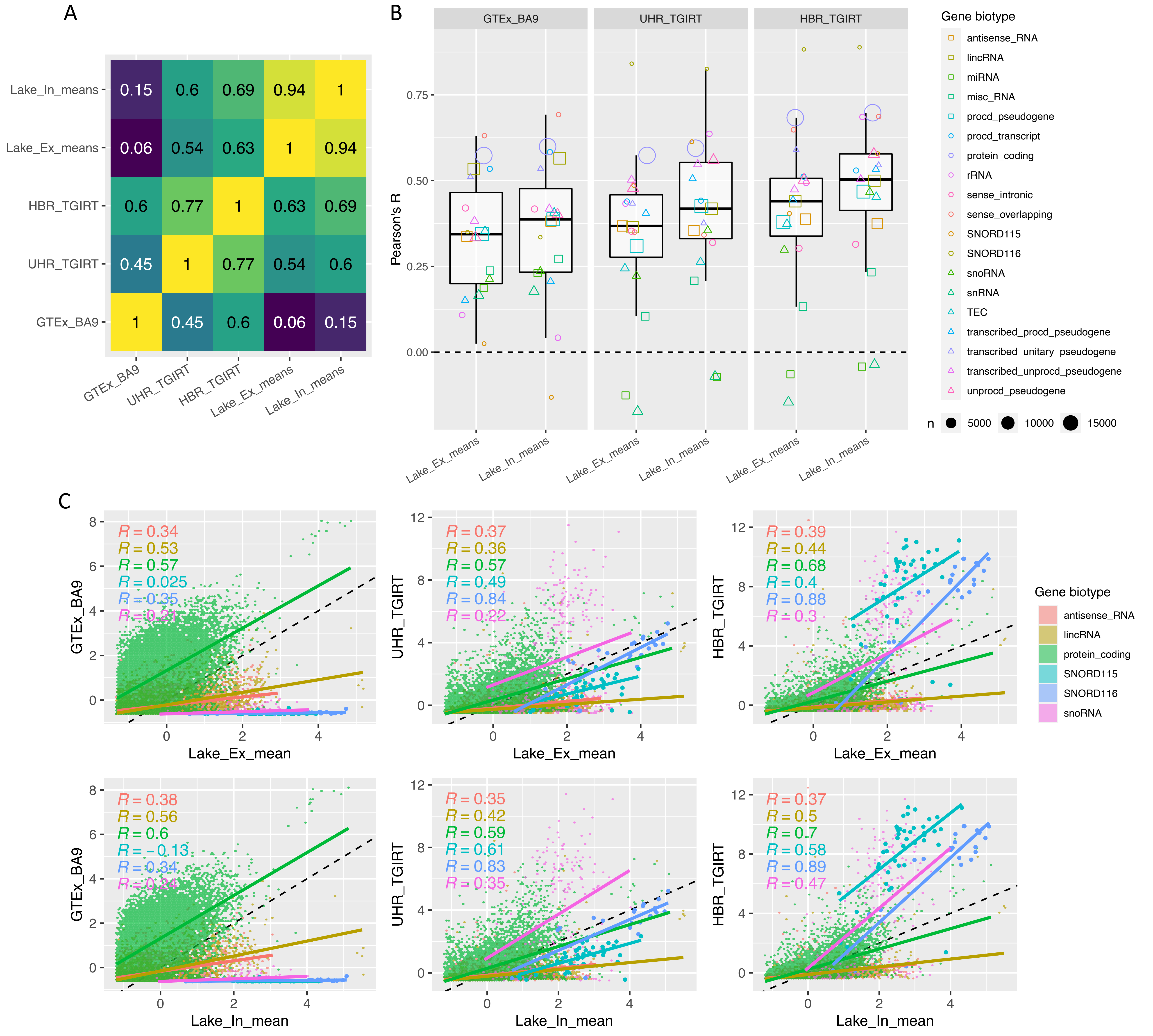

SFigure 7. Single-nucleus SMART-seq resembles unbiased TGIRT-based transcriptome quantification. A) Spearman rank correlations between transcript abundances averaged across sNuc-seq excitatory and inhibitory neurons (Lake_In/Ex_means), GTEx frontal brain cortex bulk RNAseq (GTEx_BA9), the Universal Human Reference RNA quantified via TGIRT (UBR_TGIRT), and Human Brain Reference TGIRT (HBR_TGIRT). B) Pearson correlations for different biotypes contribute to higher overall correlation between Lake et al sNuc-seq samples and HBR_TGIRT. SNORD116 cluster genes show the highest correlation between TGIRT and sNuc-seq data. C) Scatter plots of genes grouped into hexagonal bins, coloured by biotype, comparing Lake et al sNuc-seq samples, GTEx and TGIRT samples. Pearson's R values are displayed at top left. Whereas snoRNA genes are mostly undetected in GTEx_BA9, these genes show strong expression and correlation between Lake et al sNuc-seq samples and TGIRT samples, especially TGIRT_HBR.
