# Supplementary material for: A survey of RNA editing at single-cell resolution links interneurons to schizophrenia and autism
Source: RNA. 2021 Dec;27(12):1482–96. doi: 10.1261/rna.078804.121 (PMC8594476; doi:10.1261/rna.078804.121)
Supplement: Supplemental Material [file supp_078804.121_Supplementary_Methods.docx]

# Supplementary Methods

Differentially edited sites reported in neuropsychiatric brain analyses [(Tran et al. 2019; Breen et al. 2019)](https://paperpile.com/c/m9Hfcf/NlFqW+luDKc), were downloaded, and where required sites were transposed from Hg19 to Hg38 using liftOver software [(Hinrichs et al. 2006)](https://paperpile.com/c/m9Hfcf/DZPgb). Genomic ranges for Alu and non-Alu repeats in Hg38 (RepeatMasker track) were downloaded from the UCSC Table Browser [(Kent et al. 2002)](https://paperpile.com/c/m9Hfcf/7SNd2); common human single nucleotide polymorphisms were downloaded from dbSNP (GRCh38p7; build 151) [(Sherry et al. 2001)](https://paperpile.com/c/m9Hfcf/3iwRo). Genes with known amino acid re-coding sites in the brain; those implicated in autism spectrum disorder (SFARI database); those with extremely low genomic mutation tolerance, and targets of RBFOX splicing, were retrieved from tables provided in the relevant literature [(Nishikura 2016; Abrahams et al. 2013; Weyn-Vanhentenryck et al. 2014; Samocha et al. 2014)](https://paperpile.com/c/m9Hfcf/qfPMe+M3CDE+1llx6+0GDAC). Predicted binding/interacting partners for small nucleolar RNAs were curated from snoDB and studies reporting differential expression upon snoRNA transfection [(Bouchard-Bourelle et al. 2020; Kishore et al. 2010; Falaleeva et al. 2015; Soeno et al. 2010)](https://paperpile.com/c/m9Hfcf/MUzmL+FgsM2+ZyPJd+3HCzE). Transcripts Uniprot domains predicted to encompass editing-induced amino acid substitutions were retrieved based on Ensembl gene IDs from <https://www.uniprot.org/uploadlists/>.

**Supplementary References**

[Abrahams, Brett S., Dan E. Arking, Daniel B. Campbell, Heather C. Mefford, Eric M. Morrow, Lauren A. Weiss, Idan Menashe, Tim Wadkins, Sharmila Banerjee-Basu, and Alan Packer. 2013. “SFARI Gene 2.0: A Community-Driven Knowledgebase for the Autism Spectrum Disorders (ASDs).”](http://paperpile.com/b/m9Hfcf/M3CDE) *[Molecular Autism](http://paperpile.com/b/m9Hfcf/M3CDE)* [4 (1): 36. https://doi.org/](http://paperpile.com/b/m9Hfcf/M3CDE)[10.1186/2040-2392-4-36](http://dx.doi.org/10.1186/2040-2392-4-36)[.](http://paperpile.com/b/m9Hfcf/M3CDE)

[Bouchard-Bourelle, Philia, Clément Desjardins-Henri, Darren Mathurin-St-Pierre, Gabrielle Deschamps-Francoeur, Étienne Fafard-Couture, Jean-Michel Garant, Sherif Abou Elela, and Michelle S. Scott. 2020. “snoDB: An Interactive Database of Human snoRNA Sequences, Abundance and Interactions.” *Nucleic Acids Research* 48 (D1): D220–25. https://doi.org/](http://paperpile.com/b/m9Hfcf/MUzmL)[10.1093/nar/gkz884](http://dx.doi.org/10.1093/nar/gkz884)[.](http://paperpile.com/b/m9Hfcf/MUzmL)

[Breen, Michael S., CommonMind Consortium, Amanda Dobbyn, Qin Li, Panos Roussos, Gabriel E. Hoffman, Eli Stahl, et al. 2019. “Global Landscape and Genetic Regulation of RNA Editing in Cortical Samples from Individuals with Schizophrenia.” *Nature Neuroscience*. https://doi.org/](http://paperpile.com/b/m9Hfcf/luDKc)[10.1038/s41593-019-0463-7](http://dx.doi.org/10.1038/s41593-019-0463-7)[.](http://paperpile.com/b/m9Hfcf/luDKc)

[Falaleeva, Marina, Justin Surface, Manli Shen, Pierre de la Grange, and Stefan Stamm. 2015. “SNORD116 and SNORD115 Change Expression of Multiple Genes and Modify Each Other’s Activity.” *Gene* 572 (2): 266–73. https://doi.org/](http://paperpile.com/b/m9Hfcf/ZyPJd)[10.1016/j.gene.2015.07.023](http://dx.doi.org/10.1016/j.gene.2015.07.023)[.](http://paperpile.com/b/m9Hfcf/ZyPJd)

[Hinrichs, A. S., D. Karolchik, R. Baertsch, G. P. Barber, G. Bejerano, H. Clawson, M. Diekhans, et al. 2006. “The UCSC Genome Browser Database: Update 2006.” *Nucleic Acids Research* 34 (Database issue): D590–98. https://doi.org/](http://paperpile.com/b/m9Hfcf/DZPgb)[10.1093/nar/gkj144](http://dx.doi.org/10.1093/nar/gkj144)[.](http://paperpile.com/b/m9Hfcf/DZPgb)

[Kent, W. James, Charles W. Sugnet, Terrence S. Furey, Krishna M. Roskin, Tom H. Pringle, Alan M. Zahler, and David Haussler. 2002. “The Human Genome Browser at UCSC.” *Genome Research* 12 (6): 996–1006. https://doi.org/](http://paperpile.com/b/m9Hfcf/7SNd2)[10.1101/gr.229102](http://dx.doi.org/10.1101/gr.229102)[.](http://paperpile.com/b/m9Hfcf/7SNd2)

[Kishore, Shivendra, Amit Khanna, Zhaiyi Zhang, Jingyi Hui, Piotr J. Balwierz, Mihaela Stefan, Carol Beach, Robert D. Nicholls, Mihaela Zavolan, and Stefan Stamm. 2010. “The snoRNA MBII-52 (SNORD 115) Is Processed into Smaller RNAs and Regulates Alternative Splicing.” *Human Molecular Genetics* 19 (7): 1153–64. https://doi.org/](http://paperpile.com/b/m9Hfcf/FgsM2)[10.1093/hmg/ddp585](http://dx.doi.org/10.1093/hmg/ddp585)[.](http://paperpile.com/b/m9Hfcf/FgsM2)

[Nishikura, Kazuko. 2016. “A-to-I Editing of Coding and Non-Coding RNAs by ADARs.” *Nature Reviews. Molecular Cell Biology* 17 (2): 83–96. https://doi.org/](http://paperpile.com/b/m9Hfcf/qfPMe)[10.1038/nrm.2015.4](http://dx.doi.org/10.1038/nrm.2015.4)[.](http://paperpile.com/b/m9Hfcf/qfPMe)

[Samocha, Kaitlin E., Elise B. Robinson, Stephan J. Sanders, Christine Stevens, Aniko Sabo, Lauren M. McGrath, Jack A. Kosmicki, et al. 2014. “A Framework for the Interpretation of de Novo Mutation in Human Disease.” *Nature Genetics* 46 (9): 944–50. https://doi.org/](http://paperpile.com/b/m9Hfcf/0GDAC)[10.1038/ng.3050](http://dx.doi.org/10.1038/ng.3050)[.](http://paperpile.com/b/m9Hfcf/0GDAC)

[Sherry, S. T., M. H. Ward, M. Kholodov, J. Baker, L. Phan, E. M. Smigielski, and K. Sirotkin. 2001. “dbSNP: The NCBI Database of Genetic Variation.” *Nucleic Acids Research* 29 (1): 308–11. https://doi.org/](http://paperpile.com/b/m9Hfcf/3iwRo)[10.1093/nar/29.1.308](http://dx.doi.org/10.1093/nar/29.1.308)[.](http://paperpile.com/b/m9Hfcf/3iwRo)

[Soeno, Yuuichi, Yuji Taya, Taras Stasyk, Lukas A. Huber, Takaaki Aoba, and Alexander Hüttenhofer. 2010. “Identification of Novel Ribonucleo-Protein Complexes from the Brain-Specific snoRNA MBII-52.” *RNA*  16 (7): 1293–1300. https://doi.org/](http://paperpile.com/b/m9Hfcf/3HCzE)[10.1261/rna.2109710](http://dx.doi.org/10.1261/rna.2109710)[.](http://paperpile.com/b/m9Hfcf/3HCzE)

[Tran, Stephen S., Hyun-Ik Jun, Jae Hoon Bahn, Adel Azghadi, Gokul Ramaswami, Eric L. Van Nostrand, Thai B. Nguyen, et al. 2019. “Widespread RNA Editing Dysregulation in Brains from Autistic Individuals.” *Nature Neuroscience* 22 (1): 25–36. https://doi.org/](http://paperpile.com/b/m9Hfcf/NlFqW)[10.1038/s41593-018-0287-x](http://dx.doi.org/10.1038/s41593-018-0287-x)[.](http://paperpile.com/b/m9Hfcf/NlFqW)

[Weyn-Vanhentenryck, Sebastien M., Aldo Mele, Qinghong Yan, Shuying Sun, Natalie Farny, Zuo Zhang, Chenghai Xue, et al. 2014. “HITS-CLIP and Integrative Modeling Define the Rbfox Splicing-Regulatory Network Linked to Brain Development and Autism.” *Cell Reports* 6 (6): 1139–52. https://doi.org/](http://paperpile.com/b/m9Hfcf/1llx6)[10.1016/j.celrep.2014.02.005](http://dx.doi.org/10.1016/j.celrep.2014.02.005)[.](http://paperpile.com/b/m9Hfcf/1llx6)
